# Effects of exoskeletal gait assistance on the recovery motion following tripping

**DOI:** 10.1371/journal.pone.0229150

**Published:** 2020-02-24

**Authors:** Yasuhiro Akiyama, Yusuke Fukui, Shogo Okamoto, Yoji Yamada

**Affiliations:** Department of Mechanical Systems Engineering, Nagoya University, Nagoya, Aichi, Japan; Beijing University of Posts and Telecommunications, CHINA

## Abstract

Physical assistant robots improve the user’s ability to walk. However, they also potentially affect recovery motion following tripping. The assist algorithm should not interfere with the recovery motion, and should enhance the ability of the user to recover after tripping. Thus, in this study, we investigated the recovery motion affected by the assist robot after tripping. We compared the recovery motion with different reaction algorithms. Principal component analysis revealed the effects of the reaction algorithm. Correspondingly, principal components were related to the recovery motion during two steps following tripping. Specifically, the effects of the reaction algorithm were related to a principal component that represented the motion of the second step after tripping and that increased the margin of stability. Furthermore, the margin of stability became significantly large when the assistive torque was applied during the recovery motion. The result of this study suggests that the assist robot can potentially enhances the recovery motion of its user following tripping.

## Introduction

Physical assistant robots are now associated with a broad range of applications in various facets of daily lives compared to their initial uses for rehabilitation in hospitals [1, 2]. Therefore, assist robots are required to improve their configurations and algorithms to fit the new environments. In particular, the fundamental problem associated with the introduction of the assist robot in daily living environments pertains to their abilities to become accustomed with the variety and flexibility of motion types [3]. Accordingly, the expansion of the range of uses and types of environments concomitantly generates new types of risks for assist robots [4].

In the case of the gait assist robot, the risk of falling becomes critical from the viewpoint of the product’s liability. Even in daily living environments, humans can naturally fall by themselves because they occasionally trip or slip [5, 6]. If a motion mismatch occurs between the assist robot and its user during the recovery motion to avoid the fall, the risk of falling will probably increase. Thus, it is required that the assist robot does not disturb the reaction motion induced by perturbations [7]. Furthermore, it is better to enhance the recovery motion if this is possible. For this purpose, the evaluation and quantification of the effects of the assistant robot on the recovery motion is required.

The motion characteristics associated with the avoidance of the fall have been studied for many years from the viewpoint of human gait. The two major strategies adopted against tripping during gait are the “elevating” and “lowering” strategies [8]. When tripped in the early to the middle parts of the swing phase, humans frequently overcome the obstacle by lifting the tripped leg [9, 10]. This is referred to as the elevating strategy. By contrast, when the tripping occurs in the middle to the late parts of the swing phase, humans typically place the tipped leg behind the obstacle and step the opposite leg forward. This is commonly referred to as the lowering strategy. Although it is likely that this strategy will be adopted even when an assist robot is used, this assertion has not been verified yet.

Furthermore, to evaluate the stability of the posture and motion, many methods have been proposed [11]. Some of these indices are designed to evaluate the periodicity of the gait motion. However, they are not suitable for the evaluation of the reaction against large perturbation because the recovery motion largely differs from the periodic gait motion. Other methods have been based on the consideration of dynamic stability [12, 13]. However, it is not easy to evaluate the absolute stabilities of gait and recovery motion using the dynamic stability index because these types of motions are not inherently physically stable. Therefore, these indices should be used as the relative measures of the degree of imbalance of the motion. These indices are probably applicable for the evaluation of the recovery motion.

Therefore, the measurement and evaluation of the recovery motion following tripping could help improve the utility of the assist robot. Specifically, the effect of the assist robot on the performance of the recovery motion is important. Therefore, in this study, we hypothesized that the reaction algorithm that is based on the estimation of the torque applied by an assist robot during the recovery motion affects the ability to recover following tripping. The effects of the reaction algorithm could be evaluated by comparing the recovery motions between different reaction algorithms. Furthermore, the characteristics of the recovery motion in the case of a human robot user could be identified. These are the numerous issues that have not been considered nor investigated in an exhaustive manner.

## Methods

The experiments were performed with the permission of the institutional review board of Nagoya University (approval number: 17–2). Before the onset of the experiment, the subjects provided informed consent and signed the relevant consent forms.

### Apparatus

#### Assistive devise

**Fig 1:**
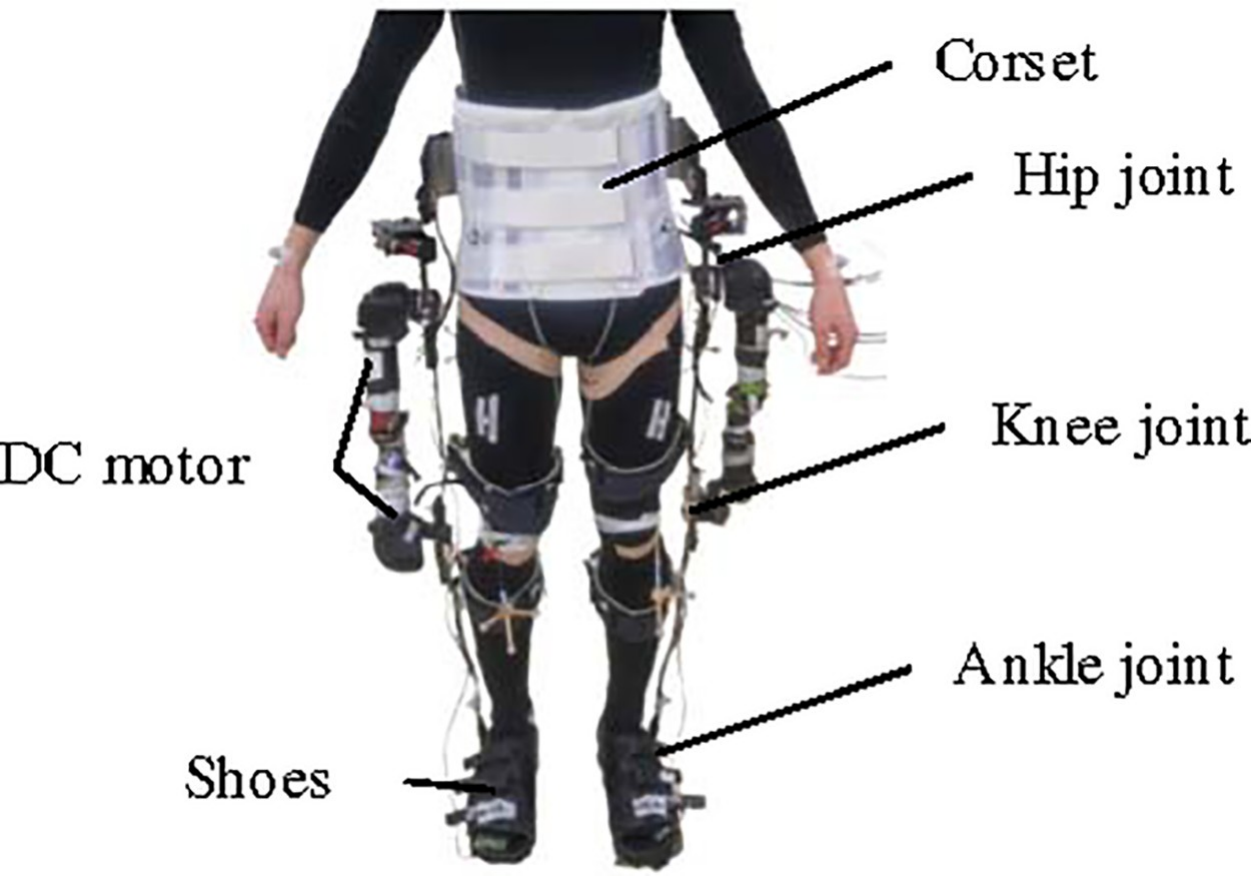
Structure of the motor actuated lower-limb orthosis (MALO) exoskeleton corset, frames, and shoes with single degree-of-freedom rotation joints. Hip and knee joints are equipped with direct current motors.

A lower-leg exoskeleton which was developed in our lab, referred to as motor actuated lower-limb orthosis (MALO), was used in this experiment. It consisted of a corset, thigh frames, shank frames, and shoes, as shown in Fig 1. Each neighboring link was connected by a single degree-of-freedom (DOF) rotation joint. Hip and knee joints were actuated using direct current motors (RE 40, Maxon Motor AG, Sachseln, Switzerland). The corset was made of flexible plastic to fit the wearer. A belt was used to fix the frames of MALO to the thigh and to the shank of the user. MALO’s weight was supported by itself through a robotic frame. Four mobile force plates (M3D, Tec Gihan Co., Ltd., Kyoto, Japan), which could measure 6-DOF forces and torques, were fixed on the sole. They were placed under the toes and heels of both feet. Heel contact (HC) was determined at the time at which the normal component of the ground reaction force (GRF)—as measured by the force plates—exceeded 100 N.

The mean time duration of the previous two gait cycles (GCs) was assumed as the gait duration of the subsequent GC. The GC was determined as the time period between the successive heel contacts of one of the two legs. The current gait phase was calculated as the ratio of the time from the preceding HC and the estimated gait duration.

The gait assist algorithm of normal walking was designed to help the forward movement of the body during the stance phase and the leg swing during the swing phase. At the stance phase, the hip joint was assisted toward the direction of leg extension and occurred during the GC (15–45% interval). Furthermore, knee flexion was assisted during the 30–60% interval of the GC. At the swing phase, hip flexion was assisted during the 65–95% interval of the GC. The knee extension assist was applied during the 75–95% interval of the GC. The maximum assistive torque was set at 7 Nm, and corresponded to 20–25% of the inner torque of the lower limb joints [14, 15]. The upward and downward edges of the assistive torque pattern were smoothed to achieve comfortable assist using a square sinusoidal function.

Furthermore, the control algorithm of MALO was designed to compensate for the friction torques of all the joints which were measured a priori. Furthermore, the assistive torque was interrupted when the joint started rotating toward the inverse direction to that of the assistive torque to prevent interference with the user’s motion, even at the time instant at which the assist torque should have been applied.

When the user tripped, there was an option to completely stop the application of the assist torque. The only exception was the torque which was applied for friction compensation. This was referred to as the “Stop pattern.” This constituted another option for the continuous application of assist torque that was designed for normal gait patterns. This was referred to as the “Continue pattern.” The former pattern was based on the consideration that the robot should not interfere with the reaction motion of the user in emergency cases. However, the implementation of this strategy is possible only when the tripping could be detected accurately online. Subsequent pattern is easily installed because the modification or switching from normal assist pattern is not required. However, the assist torque probably affects the recovery motion although our algorithm was designed in such a way that the torque was not applied to the inverse direction of the joint rotation. These reaction algorithms were designed as the basic algorithmic tools to evaluate the feasibility and effectiveness of the gait assist process.

#### Experimental setup

**Fig 2:**
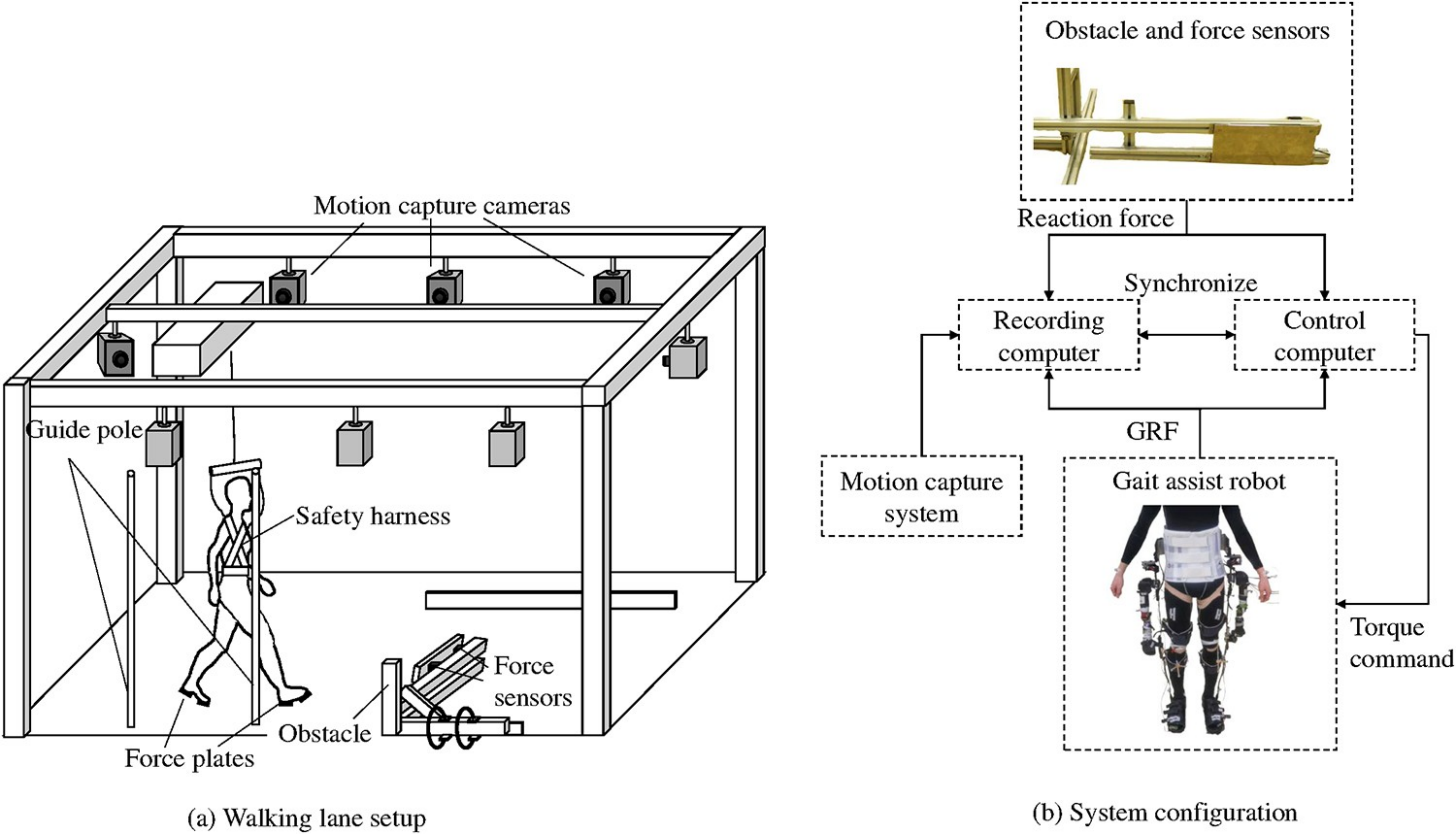
Experimental setups. (a) The subject is tripped by an obstacle in the middle of the walking lane. A safety harness was connected to the gondola to support the subject in the cases of falls. Gait timing is controlled using a guide pole and metronome. (b) The reaction force of the obstacle was used to detect tripping and switch the assist pattern to reaction algorithms. The assist torque was applied based on the gait cycle estimated from the timing of gait events.

The overview of the experimental setup is shown in Fig 2. The walking and tripping experiment was conducted in a walking lane with dimensions of 5 × 8 m (W × L). Part of this lane (4 × 5 m) was used as the acceleration and deceleration area. In the middle of the walking lane, the subject who wore the MALO was forced to trip occasionally with the use of an obstacle which was fixed on the ground, as shown in Fig 2(a).

The gait and recovery motions following tripping were recorded using 10 camera motion capture system (MAC 3D System, Motion Analysis Corporation, Rohnert Park, CA, US). To observe the motion of the subject, markers were attached on 24 feature points on the human volunteers. The selected feature points were as follows: top head, front head, rear head, sternum, shoulders, elbows, wrists, anterior superior iliac spines (ASISs), posterior superior iliac spines (PSISs), greater trochanters, knees, ankles, toes, and heels. For some points covered or hidden by MALO, cluster markers were used.

The subject wore a safety harness which connected the gondola placed above the walking lane and which moved with the subject to prevent falls. The cable of the harness supported the subject using air spring but it was usually slacked to avoid interference with the gait motion. In addition, protectors and supporters were attached to the subject.

The obstacle which was used to trip the subject consisted of a metal plate, as shown in Fig 2(b). The height of the obstacle was 15 cm, and was within the range of obstacles used in tripping experiments [16, 17]. The position of the obstacle was adjusted for each subject so that tripping occurred in the early swing phase.

The timing of tripping and the impact force were measured using four force sensors (M3D-EL-FP-U, Tec Gihan Co., Ltd., Kyoto, Japan) which were fixed behind the tripping plate. As shown in Fig 2(b), the force applied to the obstacle was monitored by the computer which executed the assist algorithm and implemented the switching process. The threshold of the tripping was set to 60 N. Although this tripping detection scheme cannot be used outside the laboratory, we selected this method to concentrate on the analysis of the effects of the reaction algorithm.

To prevent the subjects from anticipating the tripping, the subjects wore goggles which covered their lower halves. Furthermore, to stabilize the gait of the subject in all the trials, especially after the tripped trials, two guide poles were placed on the right side of the stepping position of the acceleration area as indicators. In addition, a mark was placed at the extension of the walking direction to maintain the walking pattern along a straight line.

### Protocol

Ten healthy male subjects participated in the experiment. The average age was 23.1 years, and the standard deviation (SD) was 1.8. Their mean height and weight were 172.9 cm (± 3.5 SD), and 63.6 kg (± 5.1 SD), respectively.

Subsequently, the subjects wore well-fitted sportswear, the MALO, safety gears, and reflective markers, to allow the recording of the motion trajectories. To adapt to MALO, the subject walked on the walking lane repeatedly until the gait motion became stable. The positions of the guide pole and the obstacle were determined at the end of this training session. The tripping experiment then began. The subject was allowed to take breaks as needed.

In each trial, the subject stepped in place to initiate gait and adjusted the assist timing before he moved along the walking lane. The subject then started to walk forward along the lane to the beat of the metronome and in compliance with the foot indicator.

As mentioned above, one of the two reaction algorithms applied the stop and continue patterns to the MALO after tripping. The reaction algorithm was selected randomly according to the trial. At the same time, the side at which tripping occurred was changed to eliminate the effects of the anticipation of tripping. Furthermore, dummy control trials were conducted during which no tripping occurred.

In total, 30 trials were conducted for each subject which included 10 dummy trials. The number of trials of each condition was controlled and was maintained the same. Furthermore, the side of tripping (i.e., the selection of the tripping leg) was randomized. Accordingly, the dummy trial was randomly inserted in the sequence of conducted trials.

### Data processing

The motion of the subject, GRF, and the interaction force at the obstacle, were acquired at 100 Hz. The recorded motion was smoothed using a 6 Hz Butterworth filter. The joint angle, posture, and the position of the center of mass (CoM) of the subject were calculated based on the fitting of a human model to the position of the markers using the least-squares method. The biomechanical analysis software SIMM (SIMM, MulsculoGraphics Inc., Evanston, IL, US), was used for this process. The CoM of the entire body was calculated using Zatsiorsky’s method [18]. Recorded recovery motions of tripped trials were combined across subjects in the analysis process. In this study, the effects of the contact side differences were ignored.

Regarding the recovery motion following tripping, an elevating strategy was anticipated in this experiment according to previous research studies which analyzed the relationship between the phase of tripping and reaction strategy [8, 10]. Furthermore, preliminary experiments suggested that the fall avoidance motion could not be completed within a single step. Thus, in this study, the recovery motion was considered until the landing of the second step following the onset of tripping. The recovery motion was separated into two phases for analysis. The first phase was determined as the motion between the hit timing (HT) and the first step (FS), which represented the timing of the landing of the tripped leg. The second phase started from the FS and continued to the second step (SS), and was performed by the leg which was opposite to the tripped leg. The parameters, which represented the recovery motion of each phase were determined as follows:

- WS_F(S): Average walking speed of each recovery step
- StT_F(S): Time duration of each recovery step
- TrL_F(S): Normalized length of the trajectory of toe during each recovery phase
- ToH_F(S): Maximum toe height during each recovery phase
- KA_F(S): Offset maximum knee flexion angle during each recovery phase
- StL_F(S): Normalized stride length of each recovery step
- CVx_F(S): Velocity of CoM in the traveling direction at the FS or SS
- CVz_F(S): Velocity of CoM in the vertical direction at the FS or SS
- MoS_F(S): Margin of stability in the traveling direction at the FS or SS
- CPz_F(S): Normalized minimum CoM height of each recovery phase

The average walking speed was calculated as the average speed of the CoM in the traveling direction within the intervals of successive HCs. The time duration of recovery step was calculated as the time period within the time intervals of successive HCs. The normalized toe trajectory was the moving length of the toe marker of the recovery leg during each recovery phase normalized by the height of each subject. The maximum toe height and knee angle were the maximum values of the toe marker and the knee angle during each recovery phase, respectively. The knee angle was offset by the mean maximum knee flexion angle of the dummy trials of each subject. The normalized stride length was determined as the moving distance of the heel marker of the recovery leg through the recovery step in the travelling direction normalized by the height of each subject. The CoM velocities in the traveling and vertical directions were determined as the CoM speeds of each direction at the timing of each HC.

The margin of stability (MoS) is a parameter which evaluates the dynamic balance by considering the area of the base of support, the position of the CoM, and the CoM velocity [19]. In this study, the MoS was determined as the distance between the position of the toe marker of the forward foot and the extrapolated CoM (XCoM) in the traveling direction. The XCoM was calculated using the following equation [19].

$$\begin{array}{c} \begin{array}{c} XCoM=CoM+\frac{vCoM}{\sqrt{g/l}} \end{array} \end{array}$$ (1)

The *vCoM* is the velocity of CoM in the travelling direction. The symbols *g* and *l* respectively denote the gravity coefficient and CoM height. The MoS, which could be successfully used to evaluate the gait balance in the lateral direction [20], was used to compare the stability in the traveling direction for the different timings and conditions used in this study. The normalized minimum CoM height was determined as the minimum height of CoM during each recovery phase normalized by the height of each subject.

The correlations among these parameters were calculated to evaluate the relationships among them. Representative factors of reaction motion were then extracted using principal component analysis (PCA) [21]. PCA is a method which is extensively used to compress the dimensions of datasets to extract desired features from them. In the field of gait analysis, Munuz et al. used PCA to classify various gait motion trajectories [22]. The differences of all the extracted parameters and factor scores between reaction algorithms were tested using the Mann–Whitney U test [23].

Furthermore, the relationship between the contact condition and recovery motion was modeled using multiple regression analysis [24]. The MoS and each principal component were selected as the objective variables and as the indices of balance after tripping. The parameters which represented the contact condition were selected as explanatory variables. The candidate parameters of the explanatory variables were determined as follows:

- *B*_*speed*_: Body speed before HT (m/s)
- *G*_*phase*_: Gait phase at HT (%)
- *T*_*angle*_: Trunk angle at HT (°)
- *P*_*force*_: Peak contact force (N)
- *R*_*assist*_: Reaction algorithm (Stop: 0, Continue:1)

The speed of the body was determined as the average speed of the CoM from 0.1 s before tripping to the tripping in the traveling direction. The trunk angle was determined as the angle of forward tilt of the trunk axis in the sagittal plane. The trunk axis was determined as the line which connected the center of the shoulder markers and the center of ASISs and PSISs. The peak contact force was the maximum load applied to the tripping plate. The parameters of the reaction algorithms were modeled as discrete values.

To avoid multicollinearity, the correlations among the explanatory variables were checked preliminarily. Moreover, the regression model was adjusted based on the selection of parameters whose contributions were significant.

## Result

### Overview of the gait and recovery motions

In total, 200 tripped trials were conducted for ten subjects. However, there were error trials in which a) the tripping phases completely differed from the early swing phases, b) the subjects collided with the obstacles multiple times, and c) the collision was partial. These error trials were not analyzed. Thus, in total, 110 tripped trials were analyzed. Among these, 53 trials were stop patterns and 57 trials were continue patterns.

Gait parameters, such as the velocity, stride length, and cadence of normal gait were calculated from five dummy trials which were randomly extracted from each subject. The average values of the gait parameters of each subject were distributed in the ranges of 0.99–1.20 m/s (walking speed), 48.2–54.8 strides/min (cadence), and 1.16–1.42 m (stride length).

**Fig 3:**
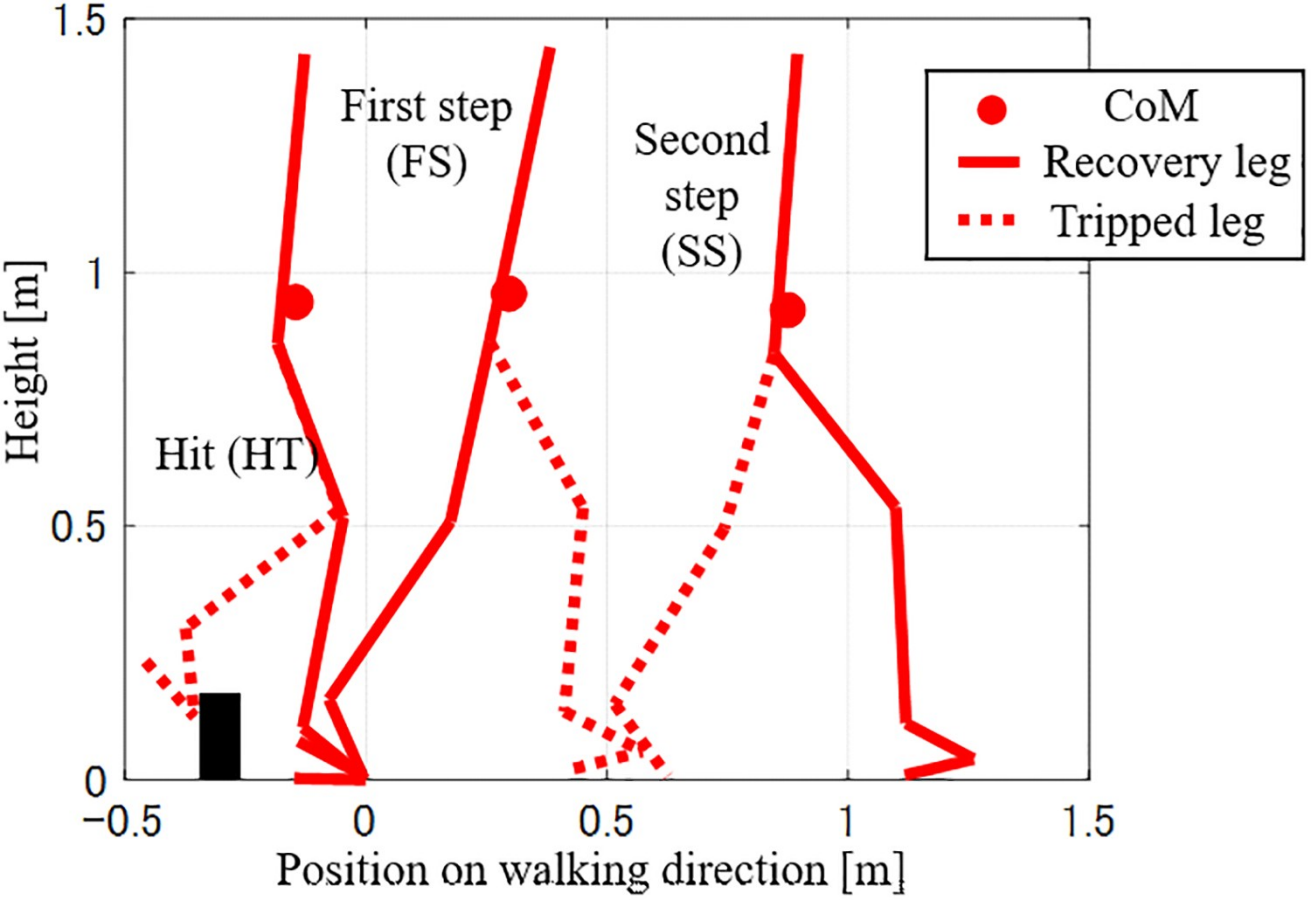
Typical reaction motion against tripping. Stick picture of the recovery motion in the sagittal plane. The left leg was tripped in this figure. The subject stepped the tripped leg forward. Then, the recovery leg was stepped.

The timing of tripping was distributed within the range of 65–80% of the GC, and corresponded to the early swing phase. In all the trials, the elevating strategy was selected. This selection was consistent with the relationship between the tripped timing and recovery strategy reported previously [10] A typical recovery motion pattern is shown and described in Fig 3. As one of the features of the elevating strategy, the tripped leg stepped forward at the FS. Furthermore, the recorded reaction motion suggested that the tripping affected the motion of the opposite step, i.e., the SS. This meant that the subject needed to take multiple steps to compensate for the effects of tripping.

### Reaction parameters

The median and interquartile range (IQR) values of recorded parameters are shown in Table 1. During the recovery motion, the subjects decelerated. Thus, WS decreased in the second phase. The large ToH and KA values in the first phase clearly indicate that the subject had to lift his foot to exceed the obstacle. Furthermore, the MoS increased in the second phase, which meant that the stability increased owing to the recovery motion. By contrast, the broad distribution of the KA values in the second phase suggests the existence of a variety of recovery motions.

**Table 1:** Median and interquartile ranges of tested parameters.

Parameter	Unit	First phase	Second phase
WS	m/s	1.03; 0.98—1.09	0.95; 0.84—1.03
StT	s	0.67; 0.64—0.76	0.55; 0.50—0.62
TrL	/height	0.67; 0.61—0.74	0.70; 0.61—0.76
ToH	m	0.32; 0.28—0.34	0.14; 0.11—0.18
KA	deg	28.11; 20.92—37.85	-0.40; -7.50—7.76
StL	/height	0.57; 0.52—0.62	0.52; 0.46—0.57
CVx	m/s	1.08; 0.98—1.20	0.96; 0.83—1.08
CVz	m/s	-0.41; -0.47 - -0.32	-0.23; -0.31 - -0.15
MoS	m	-0.09; -0.16 - -0.01	-0.01; -0.06—0.05
CPz	/height	0.53; 0.51—0.54	0.54; 0.52—0.55

The correlation matrix of these parameters is listed in Table 2. According to the definition of the MoS, it is obvious that the MoS is highly negatively correlated to the WS. However, StL is not correlated to MoS although the stride length is probably related to the expansion of the base of support. Instead, StT, which is the duration of the recovery step, is more correlated to the MoS. Although rapid and large steps tend to help avoid falls [25, 26], the same trend was not identified in this experiment. It is likely that the lengthy step time was accompanied by a sufficient step length in this experiment because subjects were sufficiently strong.

**Table 2:** Correlation coefficients of studied parameters.

	WS_F	StT_F	TrL_F	ToH_F	KA_F	StL_F	CVx_F	CVz_F	MoS_F	CPz_F	WS_S	StT_S	TrL_S	ToH_S	KA_S	StL_S	CVx_S	CVz_S	MoS_S	CPz_S
WS_F		-0.38	-0.08	-0.10	-0.30	0.14	0.76	-0.25	-0.62	0.00	0.41	-0.47	0.25	0.24	0.07	0.16	0.23	-0.15	-0.23	0.04
StT_F			0.43	0.18	0.60	0.50	0.05	-0.34	0.38	0.14	-0.02	0.28	0.32	-0.34	-0.07	0.24	-0.11	0.32	0.32	0.31
TrL_F				0.62	0.42	0.39	0.15	-0.34	0.35	0.23	-0.08	0.24	0.16	-0.24	-0.09	0.08	-0.23	0.28	0.27	0.43
ToH_F					0.59	0.09	-0.01	-0.29	0.07	0.25	-0.05	0.07	0.00	-0.27	-0.16	0.04	-0.04	0.11	0.04	0.28
KA_F						0.33	-0.04	-0.38	0.32	0.28	-0.06	0.29	0.19	-0.35	-0.25	0.19	-0.08	0.21	0.13	0.37
StL_F							0.39	-0.41	0.29	0.46	0.03	0.24	0.39	-0.06	0.04	0.50	-0.15	0.33	0.14	0.53
CVx_F								-0.35	-0.55	0.07	0.51	-0.40	0.39	0.07	0.03	0.35	0.19	-0.01	-0.14	0.23
CVz_F									-0.04	-0.14	-0.04	0.04	-0.23	0.05	-0.01	-0.15	0.12	-0.10	-0.12	-0.37
MoS_F										0.19	-0.52	0.72	0.02	-0.03	0.11	-0.01	-0.44	0.26	0.48	0.21
CPz_F											-0.04	0.06	0.08	-0.15	-0.13	0.27	-0.05	0.24	0.05	0.87
WS_S												-0.53	0.52	0.09	0.14	0.51	0.87	-0.50	-0.31	-0.06
StT_S													0.29	0.28	0.28	0.20	-0.40	0.19	0.49	0.03
TrL_S														0.47	0.51	0.87	0.46	-0.24	0.34	0.05
ToH_S															0.77	0.36	0.17	-0.51	0.11	-0.24
KA_S																0.42	0.22	-0.41	0.24	-0.21
StL_S																	0.49	-0.15	0.24	0.18
CVx_S																		-0.57	-0.38	-0.20
CVz_S																			0.19	0.35
MoS_S																				0.12
CPz_S																				

Furthermore, the CPz, which is the minimum CoM height of the second phase, was strongly correlated to the parameters of the first phase. Given that the increased CoM height helped avoid the fall, the lengthy step time and stride length of the first phase probably helped recover the body balance. At the same time, the increased CVz value negatively affected the CPz. This corresponded to an insufficient recovery process. By contrast, the effects of the parameters of the first phase on the MoS of the second phase were not so large compared to CPz.

### Principal components

**Fig 4:**
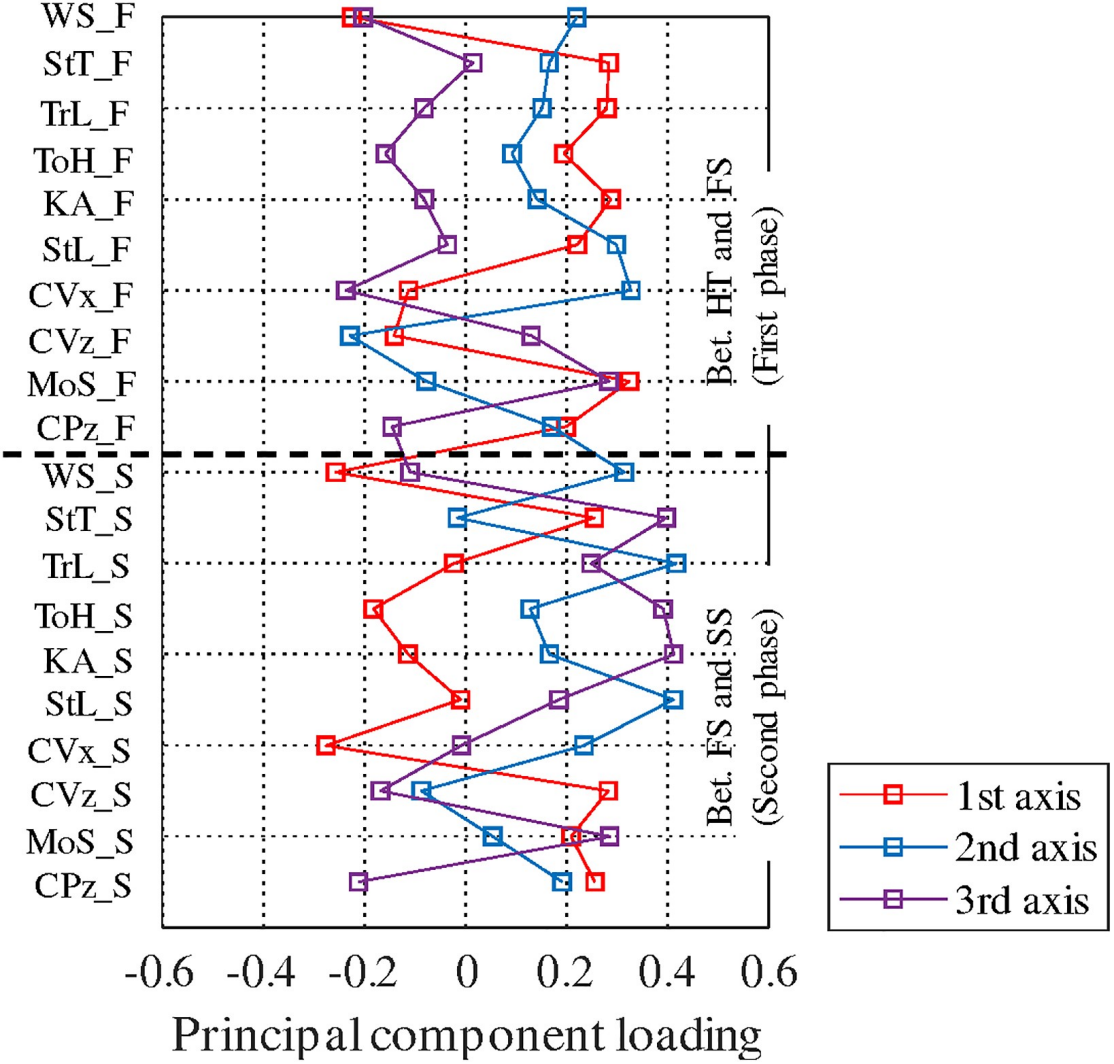
Factor loading of each principal component. The three major principal components which were associated with the respective proportions of variance of 22.3, 18.9, and 16.2%.

The factor loadings of the principal components are shown in Fig 4. The three major principal components which were associated with the respective proportions of variance of 22.3, 18.9, and 16.2%, were extracted. Thus, these major parameters explained 57.4% of the sample variance.

#### First component (early recovery)

This component is characterized by the large positive values of the parameters of the first phase. By contrast, the absolute values of the parameters in the second phase were relatively small. These features suggest that this component represents the recovery motion of the first step. Therefore, the large value of the first component corresponded to the recovery in the early phase, that is, the first phase of the recovery motion. However, it should be noted that the MoSs of both the FS and SS are positive. This means that the recovery motion of the first phase improves the balance of successive steps. Furthermore, the positive CVz and CPz values of the second phase also suggest the improvement of the balance of this phase.

#### Second component (forward movement)

This component was characterized by a positive WS, TrL, StL, and CVx, at the timing of the SS. Furthermore, the StL and CVx of the FS also contributed to this component. The movement of the subject in the traveling direction is represented by these parameters. Therefore, the large value of the second component corresponded to the forward movement during the recovery motion. In addition, the small magnitudes of the MoSs for both the FS and SS suggest that this component is not related to the recovery of balance.

#### Third component (assisted recovery)

This component is characterized by the large positive value of the second phase. It seems that this component represents the recovery motion of the second phase. However, the MoS of the first phase becomes large in value and positive. This contradiction could be explained by considering the large negative WS at the first phase. The slow body movements of the first and second phases increase the MoS according to the definition. Furthermore, as mentioned below, the value of this component increased in the “continue” trials. Therefore, this component corresponded to the recovery motion induced by the assist pattern.

### Difference of recovery motion among reaction algorithm

**Fig 5:**
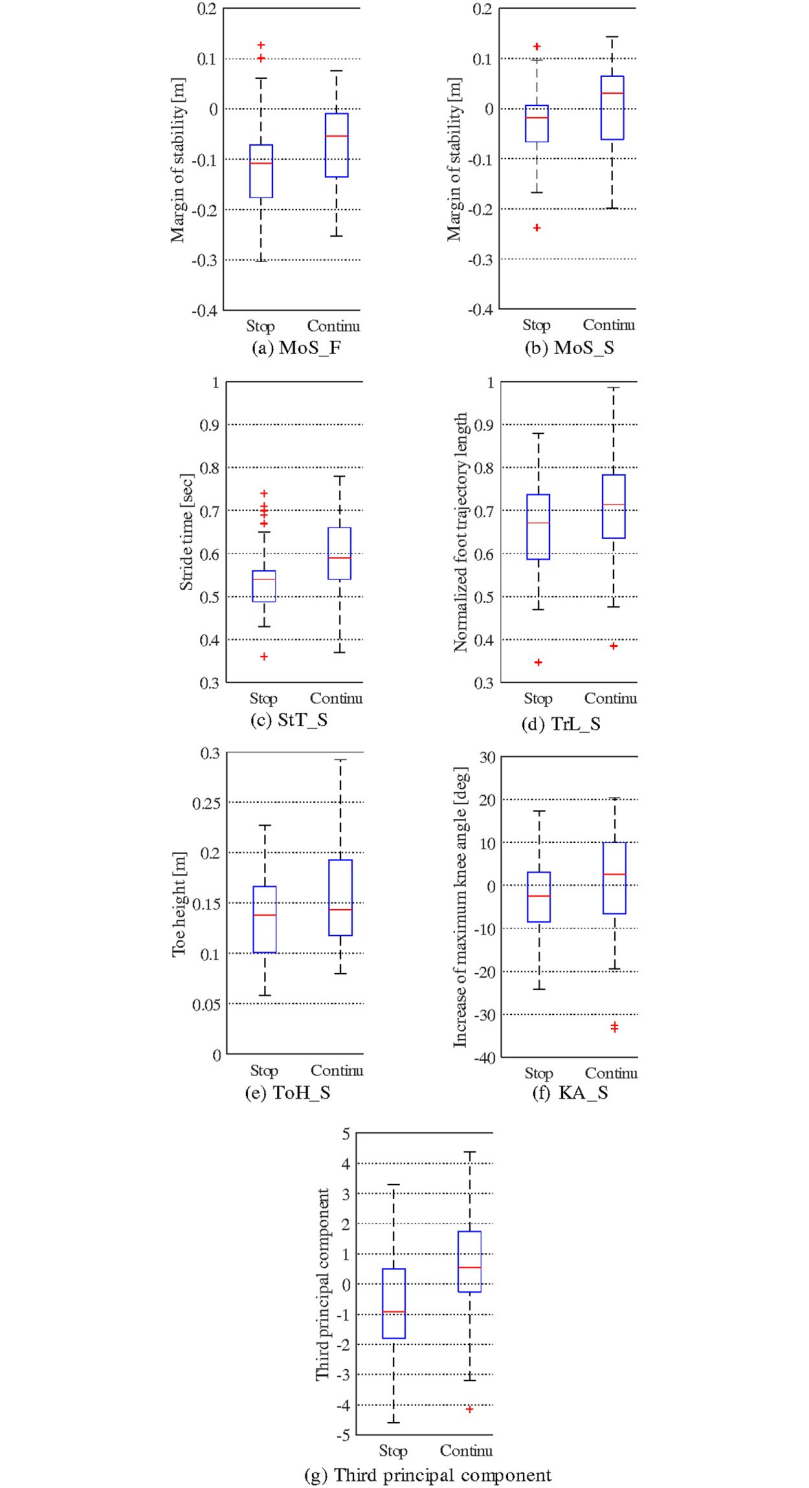
Boxplot of reaction parameters and principal component parameters which were affected by the reaction algorithm (*p* < .05) were extracted. Box represents the first and third quartiles. Outliers, which are separated from the quartile more than 1.5 times the interquartile range, are marked with crosses.

According to the Mann–Whitney U test, the reaction algorithm affected some reaction parameters and a principal component. The boxplots of these parameters are shown in Fig 5.

Among the principal components, the reaction algorithm only affected the third component. Furthermore, the parameters of the second phase were affected to a greater extent by the reaction algorithm, whereas only the MoS differed significantly among the parameters of the first step. It seems reasonable that the torque applied during the first phase affected the motion of the second phase.

### Relationship between contact condition and reaction motion

The MoS at FS could be modeled according to Eq (2) based on multiple regression analyses.

$$\begin{array}{c} \begin{array}{c} MoS=0.90-0.59B_{speed}-0.49\times 10^{-2}G_{phase}\\ -0.63\times 10^{-2}T_{angle}+0.43\times 10^{-1}R_{assist} \end{array} \end{array}$$ (2)

The ${\bar{R}}^{2}$ value of this model was 0.29 and the *p*-values of all the parameters were less than 0.01.

Although the first, second, and third components of PCA were also modeled, the *p*-values of the explanatory variables suggested that these parameters could not be explained by the parameters mentioned above.

## Discussion

### Recovery strategies and their performances

Multiple steps were required for the recovery from tripping as mentioned above. However, the ratio of the strength between the first and second steps differed among trials. The first principal component could be considered as the most representative parameter for the distinction of the recovery strategy.

**Fig 6:**
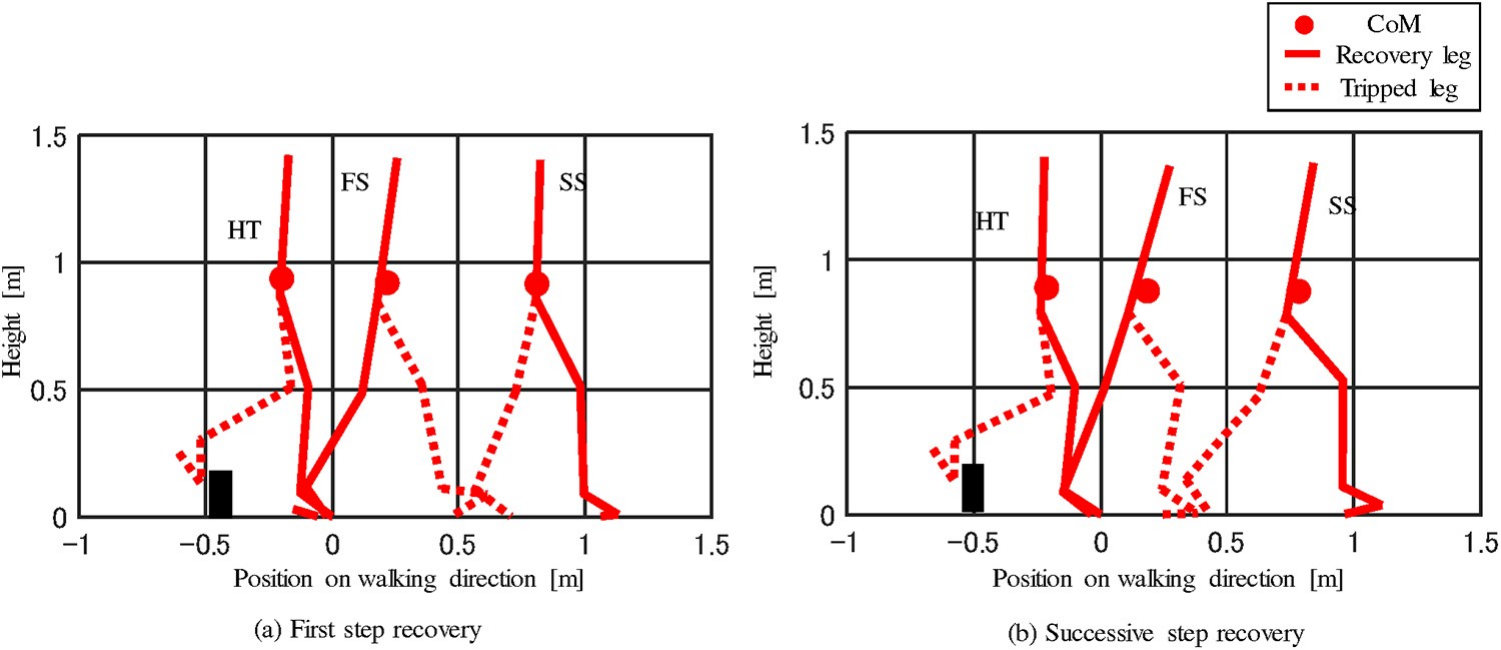
Step motion of recovery strategies. The recovery motion could be classified based on the length of recovery steps. (a) The first step after tripping was large, whereas the second step was small. (b) The first step was small and the second step was large.

In the trials whose first principal component were positive, the first step after tripping was large, whereas the second step was small, as shown in Fig 6(a). The increase of the MoSs at the FS and SS that occurred in conjunction with the increase of the first principal component, suggested that the large first step effectively recovered the gait balance. By contrast, when the first step was not sufficiently large to recover balance, the subject had to increase the step length of successive step to continue to recover balance, as shown in Fig 6(b).

Thus, from the viewpoint of balance recovery, it seemed more appropriate to place the first step at a distant location. Previous studies also suggested the effectiveness of this strategy [26, 27]. Even when the assist robot was used, this strategy was effective in improving balance.

### Effects of the reaction algorithm on the recovery motion

Among the three principal components, only the third principal component—which was related to MoS—and the gait motion of the second step, differed significantly in the comparison of the reaction algorithms, as shown in Fig 5(g). Considering the similarities of the motion patterns between the normal swing and recovery steps observed in the experiment, the assist torque applied in the recovery phase probably helped the step motion. Specifically, the applied torque during the swing phase could contribute to the increase of the foot height and step length.

However, the third principal component did not affect the motion of the FS in comparison to the SS. In the case of the elevating strategy, the FS was executed soon after the tripping. Thus, the change of motion caused by the assist torque could not become so large. By contrast, the assist torque applied in the recovery phase affected the SS because there was sufficient assist time. Additionally, even though the effects of the reaction algorithm on specific parameters of the first phase were not significant, it should be noted that the MoS of the first phase was also improved in the continue condition. This trend was consistent in the cases of the component of the third principal component and the multiple regression model.

By contrast, the first principal component did not differ between the reaction algorithms. Furthermore, although we attempted to describe the first principal component from the contact condition and the reaction algorithm using the multiple regression model, this process did not work efficiently. This result suggested that the effects of the reaction algorithm on the selection of the reaction strategy were minor. However, the direct detection of the intended motion of the wearer with the use of a sensing device, such as an electromyogram, may contribute to assist the reaction motion.

### Factors which affect the recovery of balance

It seems obvious that the posture and motion before tripping affected the recovery motion. The result of multiple regression analysis suggested that the MoS could be described from *B*_*speed*_, *G*_*phase*_, *T*_*angle*_, and *R*_*assist*_, as shown in Eq (2).

The MoS decreased when the walking speed increased as mentioned above. Thus, the effect of *B*_*speed*_ of this model seems reasonable. The model also suggested that the MoS decreased when the tripping occurred at a later phase. The CoM moved forward from the support leg as the gait phase progressed during the early to the middle parts of the swing phase (during which the tripping occurred in this study). Thus, the initial condition of the recovery motion worsened when the tripping occurred at a later timing.

The trunk angle was a posture parameter at the time of tripping. The forward tilt of the trunk possibly disturbed hip flexion. This was essential to exceed the obstacle given that it limited the range of foot lifting. In addition, the forward tilt of the trunk was also equivalent to the forward movement of the CoM. Similar to the reason provided for the tripping phase, the trunk angle made the initial condition of the recovery motion worse.

*R*_*assist*_ was one of the parameters of the reaction algorithm and represented the reaction motion after tripping whereas other parameters represented the contact condition. Considering the values of the coefficients and the ranges of all the parameters, it seemed that the degree of the effect of the reaction algorithm used in this experiment on the MoS was the same as that evoked by the other parameters.

Additionally, it should be noted that the first to the third principal components, which also represented the recovery motion, could not be modeled using the parameters of posture and motion before the tripping. This result suggested that either the effects of these parameters on the recovery motion could not be described by the simple linear model, or that there were other factors which were more essential for the description of the recovery motion.

## Conclusion

The recovery motion against tripping during assisted walking was observed and analyzed in this study. When physical assistant robots are used in the daily living environment, it is likely that the wearer may trip and fall. Accordingly, the effect of the assistive torque applied by the robot during the recovery motion was evaluated in this study.

For the in-depth understanding of the responses evoked after tripping, it is essential to consider a variety of robots and assist patterns. To maximize the contribution to improve these robots, in this study, the structure of the assist robot and the assist algorithm were set to follow the general specifications of the assist robot. Subsequently, the effects of the two types of reaction algorithms that either stopped assisting after tripping, or continued to apply assistive torques, were experimentally tested. The observed recovery motions were analyzed statistically.

The result suggested that the effects of the reaction algorithm were related to a principal component which represented the motion of the SS after tripping and increased the MoS. Furthermore, the MoS became significantly large in the cases at which the assistive torque was applied. Overall, the evoked results suggested that the assistive torque applied during the recovery motion could help the subject step forward and recover balance after tripping.

## Supporting information

S1 Dataset (CSV)

S2 Dataset (CSV)
